# “Music Has No Borders”: An Exploratory Study of Audience Engagement With YouTube Music Broadcasts During COVID-19 Lockdown, 2020

**DOI:** 10.3389/fpsyg.2021.643893

**Published:** 2021-07-08

**Authors:** Trisnasari Fraser, Alexander Hew Dale Crooke, Jane W. Davidson

**Affiliations:** Faculty of Fine Arts and Music, The University of Melbourne, Melbourne, VIC, Australia

**Keywords:** social capital, community resilience, COVID-19, social distancing, collective effervescence, music, bridging, bonding

## Abstract

This exploratory study engages with eight case studies of music performances broadcast online to investigate the role of music in facilitating social cohesion, intercultural understanding and community resilience during a time of social distancing and concomitant heightened racial tensions. Using an online ethnographic approach and thematic analysis of video comments, the nature of audience engagement with music performances broadcast via YouTube during COVID-19 lockdown of 2020 is explored through the lens of ritual engagement with media events and models of social capital. The eight case studies featured virtual choirs, orchestras and music collaborations of various genres, including classical, pop and fusion styles drawing from European, Asia Minor, South African, West African, North African, Arabic, South Asian, and East Asian cultural origins. Five overarching themes resulted from thematic analysis of video comments, including *Interaction*, *Unity*, *Resilience*, *Identity*, and *Emotion*. The paper contributes important theorisation that ritual engagement and social learning fosters intercultural understanding through engaging with music both cognitively and emotionally, which can in turn shape both individual and collective identity. Online platforms provide scope for both bonding and bridging opportunities. Community resilience is supported through the sharing of knowledge, sustaining music practice during social distancing, as well as emotional support shared among audience participants, with potential wellbeing outcomes.

## Introduction

COVID-19 is highly contagious and lethal, with a cumulative global infection rate of over 72,000,000 and more than 1,610,000 deaths from December 2019 to the time of writing in December 2020 (Dong et al., 2020). The exponential rates of infection and mortality have necessitated lockdowns in many countries, with wide-ranging social, cultural, economic and political disruptions. A rise in racist commentary, discriminatory responses and policies are acknowledged to be a threat equivalent to transmission of the virus itself, disproportionately affecting marginalized groups (Devakumar et al., 2020; Ng, 2020; Wen et al., 2020).

During COVID-19 lockdown UNESCO launched the ResiliArt movement, a series of online debates with artists and cultural workers from over sixty countries about the impact of the pandemic on cultural industries. Among recommendations emerging from this movement is the necessity to share knowledge gained during the pandemic to ensure cultural diversity is promoted and safeguarded as cultural consumption increasingly moves to digital platforms (UNESCO, 2020a). Among the objectives of UNESCO’s 2005 *Convention on the Protection and Promotion of the Diversity of Cultural Expression* is the development of cultural interaction in order to build bridges among people. UNESCO (2017) subsequently supplemented the Convention with guidelines on its implementation in the digital environment, acknowledging “the emergence of new players and new logics” (p. 2) of how content is shared in the digital sphere.

During lockdown, musicians around the world adapted their music via digital means to continue to share a diversity of cultural expression and counter information distortion about the virus (UNESCO, 2020b). The current paper uses an online ethnographic approach to investigate audience reception of music from a range of cultures shared on YouTube during the COVID-19 lockdown between April and October 2020. The research investigates whether online music engagement facilitated social cohesion, intercultural understanding and community resilience.

### Theoretical Framework

Mass migration and widespread use of global communication technology has led to renewed research interest in how to balance growing cultural diversity and social cohesion (Putnam, 2007; Abascal and Baldassarri, 2015; Healy et al., 2016). Diversity of cultural expression through music may play a role in facilitating intercultural dialog and social cohesion. This is particularly pertinent in the context of both global and local responses to transnational threats such as global pandemics. To understand the social and cultural functions of music in an online environment during a global crisis, the theoretical framework of this paper draws on an interdisciplinary literature review, including the social and cultural role of music, media events as social ritual, social capital, information diffusion, online communities, and social-ecological models of community resilience.

#### The Social Function of Music

Music is a form of expression that demonstrates cultural variation while sharing many features across cultures (Mehr et al., 2019). It has been touted as holding a key to collective identity formation through its practice and consumption (De Nora, 2000; Hesmondhalgh, 2013). Existing empirical work has led to theoretical propositions that music functions as a means of social cohesion through neurohormonal processes, communication, coordination of action, empathy, and social cognition (Dunbar, 2012; Koelsch, 2013; Clayton et al., 2020). Across cultures, social bonding and expression of cultural identity have been found to be important functions of music listening (Boer and Fischer, 2010). During times of loneliness or curtailed social interaction, music listening appears to engender social cognition, playing a role as a social surrogate (Schäfer et al., 2020). Compared to other social surrogates such as TV programs and fiction, music listening has been found more likely to lead to reminiscence of important social relationships (Schäfer and Eerola, 2020).

Music also plays a predominant role in Emile Durkheim’s idea of “collective effervescence,” where shared identity, the enhancement of collective efficacy, and emotional communion is understood to emerge from participation in large scale ritualized gatherings involving music and dance (Páez et al., 2015). Group singing has been associated with positive emotional experience, feelings of connectedness and increased group efficacy and performance (Dingle et al., 2013; Good and Russo, 2016; Slater et al., 2018). Audience participation in live music events are understood to have positive social wellbeing impacts and to facilitate social capital (Packer and Ballantyne, 2011; van der Hoeven and Hitters, 2019).

Research into how the collective social function of music gatherings translate in the absence of face-to-face encounter in digital environments is in its infancy. There is empirical evidence that virtual choirs–where people record individual performances in their own time and space that are subsequently edited together to represent a synchronous ensemble–elicit a sense of social presence for participants (Fancourt and Steptoe, 2019). However studies in this special edition suggest the virtual choir during COVID-19 lockdown was experienced by participants as a stopgap measure–not as good as a face-to-face experience, despite some positive outcomes (Daffern et al., 2021; Draper and Dingle, 2021).

Ethnographic research has also explored collective engagement with digital media events through the lens of social ritual. Applying a Durkheimian perspective, Couldry (2003) defined social ritual as habitual and formalized actions involving “transcendent,” or unifying values–for example nationalism or religious beliefs. Research has considered shared experiences such as building identity through televised cultural music and dance performance, as well as mediated self-disclosure via the internet (Couldry, 2003; Pink et al., 2016). In an online context, Couldry (2003) found the shift from traditional centralized media to a decentred online network resulted in a “multiplication of centers” (p. 191), and thus multiple networks.

#### Social Capital and Online Communities

Social capital offers a theoretical framework based on networks, while also allowing for a useful perspective on efforts to balance social integration and cultural diversity online. As a broad concept, social capital refers to the resources an individual or group has access to in their social world. Numerous scholars have contributed to development of the term including Pierre Bourdieu, Nan Lin, James Coleman and Robert Putnam. This paper will focus on Putnam’s approach, as it offers a theoretical framework based on the value of social networks and shared norms primarily at the collective level (Putnam, 2000).

Putnam’s (2000) investigation of social capital in the United States popularized research into social cohesion. He was concerned about the decline in social trust in American neighborhoods, and doubtful that the fledgling internet could adequately replace face-to-face communication in building social capital. He defined social capital as the social connections between people–the networks and associated norms of reciprocity and trustworthiness. His investigation considered *bonding capital* which reinforces exclusive identities and homogenous groups and *bridging capital* which is inclusive and encompasses diverse groups. Clusters of strong connections that characterize bonding capital have been argued to be important for the reinforcement of norms, obligations and expectations (Coleman, 1988). Conversely, “weak ties” that characterize bridging capital have been argued to be important for information diffusion, access to novel ideas and integration of broader communities (Granovetter, 1973, 1983; Burt, 2005).

The success of cultural expression in creating bridges among people is predicated on engagement between different groups. On the one hand, commentators such as Benkler (2006) and Castells (2015) have argued online networks may facilitate more diverse social connections. On the other hand, the phenomenon of “echo chambers” or “filter bubbles” suggests *homophily*, the tendency to prefer association with similar people, is further reinforced online (Pariser, 2011).

The formation of online communities has been considered from the perspective of weak ties (Chen, 2011), affiliation formed via hashtags and online discourse (Zappavigna, 2012) and communities of shared interests (Baym, 2007). Debate has ensued regarding the nature of online ties–including the values espoused by online communities and the utility of online connections. While hashtags and online discourse can signal and create affiliation online, they are not always used inclusively, with the formation of “anti-social” communities advocating racist sentiments (Kreis, 2017; Murthy and Sharma, 2019; de Saint Laurent et al., 2020). While Chen (2011) argued that sense of community online is largely imagined, in her investigation of Swedish independent music fandom, Baym (2007) characterized online connections as an ecosystem or “networked collectivism” that provided opportunities for computer mediated sharing of cultural products in real time and asynchronously.

#### Social Influence, Contagion, Homophily and Empathy

Existing social connections influence how music is shared using computer mediated communication, but access to broader networks online may accelerate information diffusion. Social learning theory (Bandura, 1971) has been used as a model to understand change in music consumption based on the social influence of others online (Dewan and Ramaprasad, 2012; Dewan et al., 2017). While the influence of proximal peers tends to dominate, popularity information available in online blogs can expose people to music consumed outside their immediate social circle (Dewan et al., 2017). It is difficult however to distinguish the role of social influence from homophily through self-selection, an observation made both in research of online music sharing (Bapna and Umyarov, 2015) and emotional contagion among YouTube audiences (Rosenbusch et al., 2019).

Rosenbusch et al. (2019) noted the video format of YouTube may be stronger in impact for emotional content compared to message-based platforms such as Facebook and Twitter. Bandura (1971) observed that televised forms of modeling are particularly effective modes for social learning. Thus, YouTube may be stronger in impact for social learning about different cultures than message based platforms, particularly through the emotional medium of music. Empirical research suggests social norms related to racial bias may be implicitly learned by audiences through non-verbal cues on television shows (Weisbuch et al., 2009). There is support, employing the same research paradigm, that affiliation with a cultural group can be strengthened by listening to culturally relevant music, an effect mediated by trait empathy and engaging in culturally relevant mental imagery (Vuoskoski et al., 2017). While this previous research holds promise for combining cultural music and visuals on YouTube for strengthening affiliation with other cultures, it does suggest that individuals high in trait empathy may be more susceptible to this effect. Those high in trait empathy have similarly been found to be more susceptible to the emotional contagion effect of music (Vuoskoski and Eerola, 2012). Rosenbusch et al. (2019) analysis of YouTube comments provided evidence of both emotional contagion and homophily among YouTube audiences. Where emotional contagion refers to the direct triggering of similar emotions through interactions with others, the authors offered cognitive empathy–the capacity to understand another person’s perspective–as an alternative explanation for the data, highlighting the difficulty of distinguishing these processes, particularly in an online environment.

#### Community Resilience in Complex Systems

Berkes and Ross (2013) proposed a model of community resilience that integrates literature regarding social-ecological systems with literature from health psychology. The former defines resilience as the capacity for the system to continually adapt to absorb disturbance and retain function. The latter regards community resilience as the capacity of the social system to come together through the exchange of knowledge and resources to work toward a communal objective in response to adversity.

The online environment may be considered a complex social-ecological system. In an online environment, the spread of information can be difficult to manage, and although decentralization of information dissemination can be seen as a democratizing force, as discussed previously, it may not always be used for prosocial ends. Furthermore, the spread of music online has been observed to be unpredictable and inequitable, with “information cascades,” where people follow the music choices made by others, fueling the exponential rise in recognition of certain music (Salganik et al., 2006). As COVID-19 demonstrates, not all contagions are positive and the homogenizing effect of information cascades represents a threat to cultural diversity of expression online.

As an emotive medium with shared and distinct cultural features, ritual engagement with music online holds promise as a way to facilitate meaningful intercultural dialog, create broader affiliations between groups and enhance collective efficacy. Community resilience is argued to be strengthened through drawing on diverse cultural identities (Grossman, 2014). However, as the online environment demonstrates homogenizing and polarizing forces, conserving a diversity of cultural expression online poses a challenge. To this end, UNESCO (2017) proposed institutional intervention. Despite observation that creativity in digital environments can circumvent institutional influence, with creators themselves becoming direct influencers of cultural production (Mishra and Henriksen, 2018), research into social movements online suggests traditional media, organizations and opinion leaders, still play an important role in communication flows and social connections via online networks (Sajuira et al., 2015; Hilbert et al., 2017).

#### Integrating a Diverse Set of Theories

To establish a theoretical framework to consider the role of online music engagement in facilitating social cohesion, intercultural understanding and community resilience during a global pandemic, a diverse set of theories has been considered. The following analysis draws mainly from a Durkheimian perspective of social integration through ritual engagement and a consideration of bonding and bridging social capital to understand cooperation within and between groups. Further consideration is given to interaction between different micro, meso and macro level processes. These include the mechanisms by which music might engender shared identity, including emotional contagion and social cognition, but also factors that influence the connections between people including processes of homophily, social learning, and information diffusion, which online appears to have both individual and institutional influences. Online connections and information sharing have implications for community resilience, as does the potential for music engagement to increase group efficacy and contribute to social wellbeing.

### Study Aim and Research Questions

Using an online ethnographic approach, the study explored how audiences engaged with music performances broadcast online via YouTube as a ritual during COVID-19 lockdown. The analysis addressed the following research questions:

• What were the ritual elements of audience engagement with music broadcasts related to the COVID-19 pandemic?
• What role did this online music ritual play in engendering shared identity, and what mechanisms (e.g., emotional contagion, social learning, homophily) were implicated?
• How did different cultural identities interact as part of online music engagement?
• What factors influenced dissemination of the videos?
• How is community resilience enacted through online music engagement?

## Methods

### Data Collection and Participants

Due to the burgeoning volume of online music performance that emerged during COVID-19, an online ethnographic approach was adopted to filter the content. This involved the first author watching and interacting with music-related YouTube videos posted on their social media feeds from April 1st to October 30th 2020, and selecting appropriate videos for further analysis. The aim was to capture the first author’s experience of interacting with this material from Australia, as a community engaged dance and music practitioner with strong online connections to others engaged in intercultural music and dance. Supporting this approach, ethics protocol approval was provided by the University of Melbourne Human Research Ethics Committee (Application ID#: 2057554.1).

YouTube audiences were regarded as participants and their comments posted in response to videos were considered data. To protect confidentiality and privacy, their data were deidentified. To avoid traceability via online search engines, specific details of music performances are omitted in publication, the cultural origins of the music referred to in general terms, and quotations are paraphrased, as per a fabrication approach to qualitative online research described by Burles and Bally (2018).

### Materials and Methods

A total of 10 videos were selected for analysis. These videos were chosen on the basis that: they contained footage of a musical performance featuring a fusion of cultural styles, or culturally diverse engagement; were filmed and uploaded during the COVID-19 pandemic; included content relating to the COVID-19 pandemic; and had video descriptions in English. This followed a purposive sampling approach, where the selection criteria were applied in order to identify case studies that best addressed the research questions. The 10 selected videos each related to one of eight case studies. Performance details and number of videos analyzed for each case study are presented in Table 1.

**TABLE 1:** Videos selected for analysis as organized by case study.

Case Study	Performance	No. of videos
1	Two performances by large scale community choir	2
2	Two remixes of a North African instrumental created in lockdown	2
3	South African song and dance to broadcast health message	1
4	North American and East Asian orchestra collaboration	1
5	World fusion music collaboration–charity raising effort	1
6	Asia Minor informal orchestra	1
7	West African/Arabic trio	1
8	West African/Western classical duo	1

Thematic analysis was used to code and organize data, providing a methodological flexibility suitable for an exploratory study (Braun and Clarke, 2006). The research is underpinned by a pragmatic philosophy that considers knowledge to be influenced by social experiences, and simultaneously constructed and real (Biesta, 2015). YouTube pages were imported as PDFs into NVivo 12 for Mac (QSR International, 1999) using NCapture.

Using this approach made all text on the YouTube page available for direct coding, including the introductory text for each video (in which the artist or organization often offers some explanation or description of the performance), as well as viewer comments. Line by line coding was used to assign comments in English to themes related to the stated research questions and to identify emergent patterns, using the NVivo manual text coding function. Thematic similarities and differences between case studies were analyzed. Coding and interpretation was refined through a process of investigator triangulation, drawing on a mixture of expertise and backgrounds in social and community psychology, sociology and critical studies (Archibald, 2015).

### The Context of Each Case Study

#### Case Study 1: Two Performances by Large Scale Community Choir

Case study 1 featured a live choir, established pre-COVID, which adapted to the online format during the pandemic using the virtual choir approach. Located in the Southern Hemisphere, the virtual choir attracted over 1000 participants from around the world to sing Western pop songs in English. Two of their performances were included in the analysis. Guide videos, instructions, and editing were coordinated centrally, allowing singers to participate with no more than a personal device and internet access. Using general information provided in the introductory texts, from the first to the second video there was a growth of approximately 50% in participation and 120% in the number of nations represented.

#### Case Study 2: Two Remixes of a North African Instrumental Created in Lockdown

An improvisation with a traditional North African instrument to the rhythm of a household appliance was remixed on two occasions. The original performance, which was broadcast from North America is not included in the analysis as the YouTube comments function was disabled. However, it is noteworthy that the shared cultural origin of the musicians and the name of the musical style was signaled by hashtag and keyword search terms in the original performance and in the two remixes included in the analysis, both of which were broadcast from Western Europe. One YouTube video remix featured the original footage, edited together with the remix artist’s own video recordings playing four additional instruments in the same cultural style. The second YouTube video remix featured the remix artist’s DJ logo superimposed over the original footage. The music was remixed in a Trap music style (sub-genre of Hip Hop) with electronic beats and samples.

#### Case Study 3: South African Song and Dance to Broadcast Health Message

Case study 3 was a performance coordinated and broadcast by an international organization, featuring a well-known singer adapting a South African pop song to communicate a health message about COVID-19. The song was sung in English. YouTube information revealed that dancers were chosen to be featured in the video after submitting their own home-recorded video performances dancing to the song. There were a large number of submissions, with 28 nationalities represented. Like case study 1, participation required only a personal digital device and access to internet, which appeared key in facilitating community engagement.

#### Case Study 4: North American and East Asian Orchestra Collaboration

The fourth case study involved a collaborative performance, by a professional orchestra located in North America and a student orchestra located in East Asia, in a Western classical style. Introductory text in the North American orchestra’s YouTube broadcast revealed the performance was an effort to adapt an ongoing joint endeavor between the orchestras to meet COVID-19 restrictions by using the virtual orchestra approach. Eighty musicians participated, fifty from the North American orchestra and thirty from the East Asian orchestra.

#### Case Study 5: World Fusion Music Collaboration–Charity Raising Effort

Case study 5 comprised 11 musicians of 10 different nationalities spanning West Africa, North Africa, South America, North America, East Asia, South Asia, Western Europe and the Caribbean. The composer of the song broadcast the video via his YouTube channel. It was sung in multiple languages, with the English version of the lyrics provided in the introductory text, along with a link for donations to an international disaster response organization. Accompanying video footage featured the musicians performing, intercut with shots of the disaster response organization’s activities in various communities around the world.

#### Case Study 6: Asia Minor Informal Orchestra

This performance featured 54 musicians, coordinated by two organizations, one located in the Northern Hemisphere, the other in the Southern Hemisphere. The orchestra performed a cover of a song in a vernacular style of music drawing on influences from Eastern Europe and Asia Minor. The song was sung in an associated language with lyrics provided in English in the introductory text. The majority (51.8%) of participants identified with a nationality associated with the musical style performed. Eight other nationalities were represented by 26.8% of the participants. The remaining 21.4% of participants identified transnationally.

#### Case Study 7: West African/Arabic Trio

Case study 7 featured a trio of musicians. According to the introductory text, the music performed was an Islamic invocation in response to current events. It was composed in West Africa by the broadcasting musician on a traditional instrument. He invited two other musicians to participate who were of Arabic descent, living in Western Europe. One musician accompanied on another instrument, the other sang in Arabic. The videos were recorded in each musicians’ home and edited together by the accompanying instrumentalist. The music was a fusion of West African, Arabic, and European styles.

#### Case Study 8: West African/Western Classical Duo

Case study 8 was a duo performed by two well-known artists. They played a cover version of a West African song, sung in the dialect of the composer, accompanied by Western classical instrumentation. The introductory text by the broadcasting musician was written in French and English, sending love, blessings and encouraging social distancing. The singer was of West African background, living in Western Europe and the accompanying musician was of East Asian background living in North America.

## Results

To investigate social interaction and engagement within case studies, first a line-by-line coding of viewer comments from all 10 videos was undertaken to ascertain common acts, sentiments or interactions. This allowed identification of indicators of the ritualized aspects of music participation, as displayed in the context of online media engagement. Identification of these ritual markers sets the basis for a subsequent examination, undertaken through the lens of Durkheim’s notions of social ritual and collective effervescence, as well as models of social capital. Following the establishment of overarching themes, a case-by case analysis identified converging and diverging themes.

### Languages Other Than English

Comments in languages other than English (LOTE), apparent for all cases, provided data about the cultural diversity of the audience. Despite the brevity of many of these comments, automated translation was not used, to avoid losing nuances in dialog identifiable only with specific cultural knowledge, an issue previously noted in intercultural research on YouTube comments (Oh, 2018).

### Explanation of Main Themes

Five overarching themes resulted from coding and analysis of the data. In order of prominence, they included *Interaction*, *Unity*, *Resilience*, *Identity*, and *Emotion*. The *Interaction* theme captured all distinct examples in which audience members were interacting with each other. This was an emergent theme that captured the unique affordances of online platforms to facilitate dialog between users. As further outlined in the analysis, this had implications for the exchange of knowledge regarding collaborative music practice, lockdown experiences and cultural knowledge. The *Unity* theme captured group responses and comments about shared experience and identity. The *Resilience* theme included comments about experiencing and responding to adversity during the pandemic. The *Identity* theme captured comments regarding cultural identity, and personal and collective identification with the music. The *Emotion* theme captured references to and expressions of emotion.

### Overview of Subthemes

Subthemes were derived directly from the data, and organized into the five themes above (see Table 2). These subthemes sought to capture the social nuances in basic text-based interactions. For example, the most common response across all case studies was a simple, positive statement directed toward either the performance (such as “Wow,” “Amazing”) or toward the performers (such as “Bravo everyone”). Such positive statements were interpreted to represent a group response, and initially coded collectively as *applause*. To account for the unique affordances that promote interaction between commenters online, with the possibility that an individual audience member’s comment may be read by performers and expanded upon by other views, those positive comments that were directed toward the performers were subsequently coded as a subtheme known as *shout outs*, distinct from *applause*, in that the former denotes *Interaction*, while the latter represents a sense of *Unity*. Another common audience reaction was signaling where they were in the world. This is analogous to the use of hashtags in signaling affiliation, in this case based on national identity, and was coded in the subtheme *where are we from?*, under the theme *Identity*.

**TABLE 2:** Themes and subthemes of thematic analysis.

Themes	Subthemes	Description	Examples
**1. Interaction**		**Comments that demonstrated active audience engagement with the performance**	
	(1.1) Shout outs (8 cases, 385 references)	Positive statements that addressed the performers, sometimes referring to performers by name	“*Thank you*” “*Bravo everyone*”
	(1.2) Conversations (5 cases, 174 references)	Comments that formed conversations between participants	“*I’ve been self-isolating for 10 days*” “*I know this is a hard time, on my own too*”
	(1.3) Requests (4 cases, 102 references)	Requests to participate and song requests	“*When is the next one and how do I participate?*”
	(1.4) Information diffusion (3 cases, 53 references)	Indications of having shared or intending to share the video; mentions of celebrity endorsements; and having found the performance via traditional media sources	“*I just shared this with my family in Denver*” “*<band/celebrity> shared this!*” “*I discovered this by watching <media source>*”
	(1.5) Appreciation for performers (5 cases, 50 references)	Complimentary comments about performers	“*Better than celebrities singing together*” “*This trio is a heavenly match*” “*Both of them! Marvelous*”
	(1.6) How did you do this? (1 case, 42 references)	Requests for technical information	“*Did you use a special app for this?*”
	(1.7) I had a great time (2 cases, 24 references)	Positive statements from participants	“*Such a joy and privilege to participate in this choir*”
**2. Unity**		**Group responses and comments about being or working together**	
	(2.1) Applause (8 cases, 438 references)	Positive statements, general support for the performances	“*Amazing*” “*Wonderful*”
	(2.2) We are in this together (2 cases, 48 references)	References to experiencing the pandemic together	“*Stay safe everyone*” “*We are in this together from every corner of the world*”
	(2.3) Humanity (3 cases, 35 references)	References to humanity and shared global identity	“*This seems like a gift to humanity*” “*It seems as if the whole world is a family*”
	(2.4) Music unites (3 cases, 18 references)	References to the role of music in uniting people	“*Music brings people together, when we’re further apart*”
**3. Resilience**		**Comments about the experience of lockdown, the healing role of music, inspiration and hope for sustained positive change**	
	(3.1) Music as a salve (6 cases, 130 references)	References to the healing/soothing nature of music	“*Such a beautiful way to keep our spirits up*” “*It’s a virtual hug for our wounded souls*”
	(3.2) Sharing lockdown experiences (3 cases, 104 references)	Comments related to being in lockdown and references to difficult times	“*I have spent 3 weeks alone*” “*We need to come together in these difficult times*”
	(3.3) Hope and change (6 cases, 88 references)	References to hope, inspiration, creativity, and wanting to sustain positive change	“*This gives me hope*” “*I hope this will make us better people in a better world*”
**4. Identity**		**Comments related to nationality or cultural identity and the cultural significance of the music**	
	(4.1) Languages other than English (8 cases, 138 references)	Languages other than English forms a subtheme indicating representation of different cultures in the audience	Languages, as detected by Google Translate, included Arabic, French, Greek, Portuguese, Spanish, Japanese, Korean, Chinese, Hindi, Nyanja, Malagasy
	(4.2) Nostalgia (4 cases, 110 references)	References to music linking to memories and references to earlier versions or composers	“*Loved this version of one of my favorite oldies*” “*My favorite song from childhood*”
	(4.3) Where are we from? (6 cases, 63 references)	References to where in the world the comment is from and acknowledgments of cultural diversity in performance	“*Greetings from Samoa*” “*Beautiful music from around the world*”
	(4.4) Cultural meaning of the music (6 cases, 20 references)	Comments about the style of music performed, or the use of traditional instruments	“*Beautiful playing on the bouzouki*” “*What dialect is she singing in?*”
**5. Emotion**		**References to and expressions of emotion**	
	(5.1) Tears (3 cases, 68 references)	References to tears and sadness	“*Couldn’t stop crying*” “*Tears*”
	(5.2) Joy (2 cases, 48 references)	References to joy, happiness and humor	“*Pure joy!*” “*I laughed so much*”
	(5.3) Mixed emotions (1 case, 30 references)	References to a combination of tears and joy	“*Happy tears*” “*Tears of joy*”
	(5.4) Touched (2 cases, 28 references)	References to feeling touched or moved and physical responses	“*So moving*” “*Gave me goosebumps*”
	(5.5) General emotions (1 case, 5 references)	The nature of the emotional response was unclear	“*That was emotional viewing*”

*Cases (*N*) = 8.*

*Interaction* subthemes included conversations, requests for information, requests to participate or indications of having participated, and comments related to the sharing of information. *Unity* subthemes comprised references to experiencing the pandemic together, shared identity and the role of music in uniting people. *Resilience* subthemes captured those comments that referred to the soothing nature of music, adversity in relation to the pandemic, and hope and positive change. *Identity* subthemes included LOTE, comments related to music evoking memories, acknowledgments of culturally diverse representation in the performances, and references to culturally specific aspects of the music. *Emotion* subthemes distinguished different emotional responses captured through comments including sadness, joy, mixed emotions and references to feeling moved or experiencing embodied responses such as chills.

**FIGURE 1:**
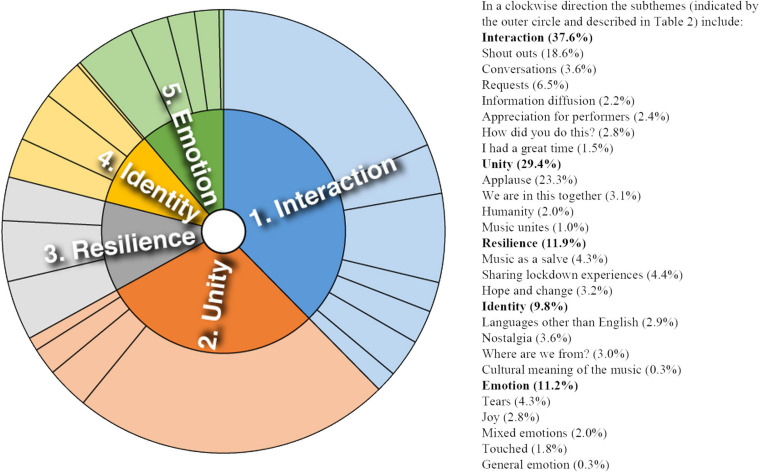
Sunburst diagram of thematic analysis for case study 1, indicating proportion of data for each theme (inner circle) and subtheme (outer circle).

**FIGURE 2:**
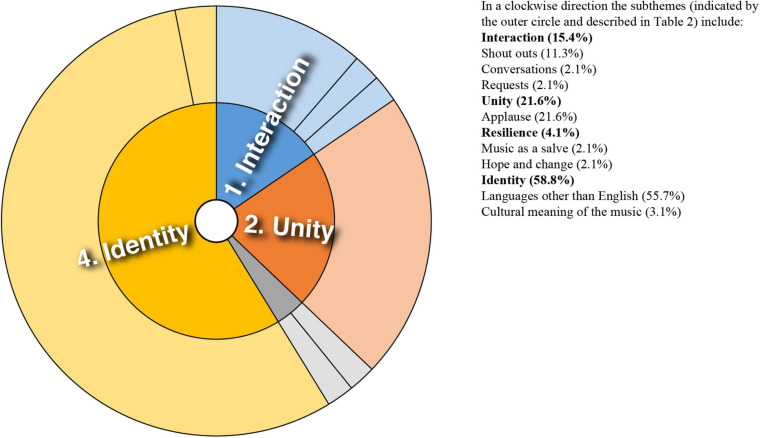
Sunburst diagram of thematic analysis for case study 2, indicating proportion of data for each theme (inner circle) and subtheme (outer circle).

**FIGURE 3:**
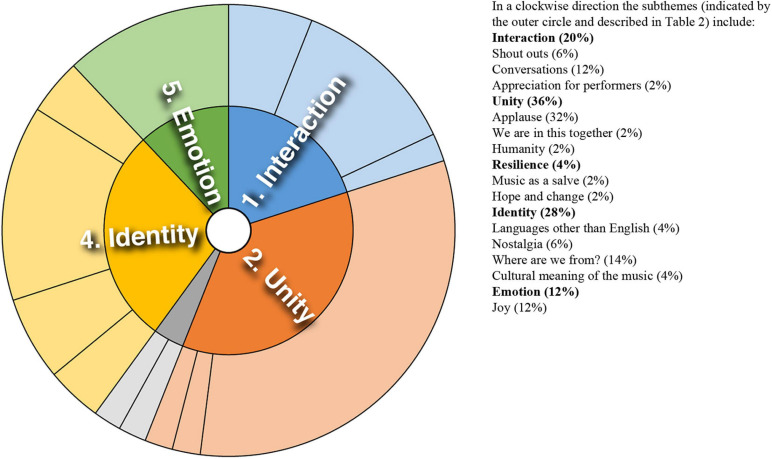
Sunburst diagram of thematic analysis for case study 3, indicating proportion of data for each theme (inner circle) and subtheme (outer circle).

**FIGURE 4:**
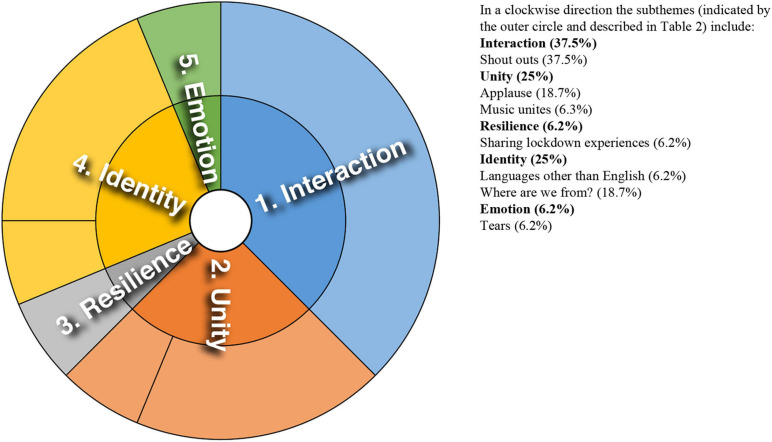
Sunburst diagram of thematic analysis for case study 4, indicating proportion of data for each theme (inner circle) and subtheme (outer circle).

**FIGURE 5:**
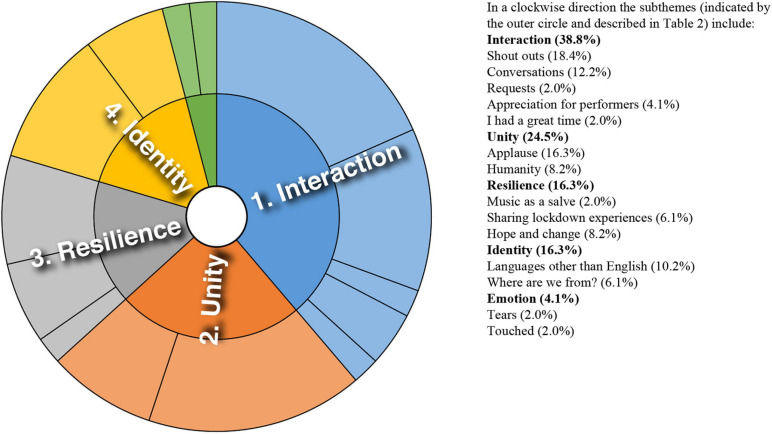
Sunburst diagram of thematic analysis for case study 5, indicating proportion of data for each theme (inner circle) and subtheme (outer circle).

**FIGURE 6:**
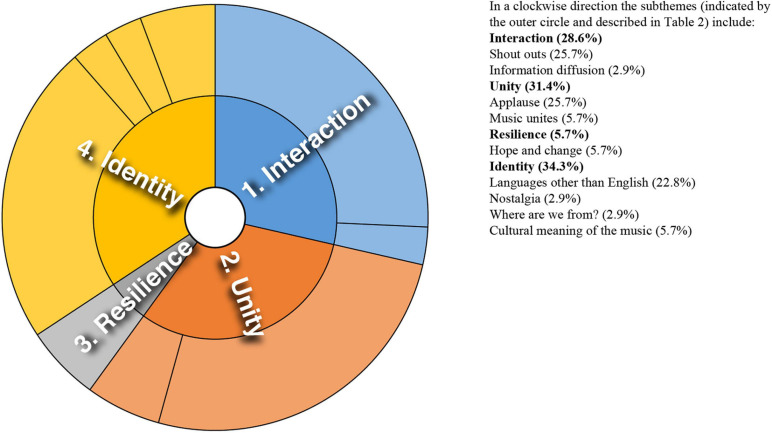
Sunburst diagram of thematic analysis for case study 6, indicating proportion of data for each theme (inner circle) and subtheme (outer circle).

**FIGURE 7:**
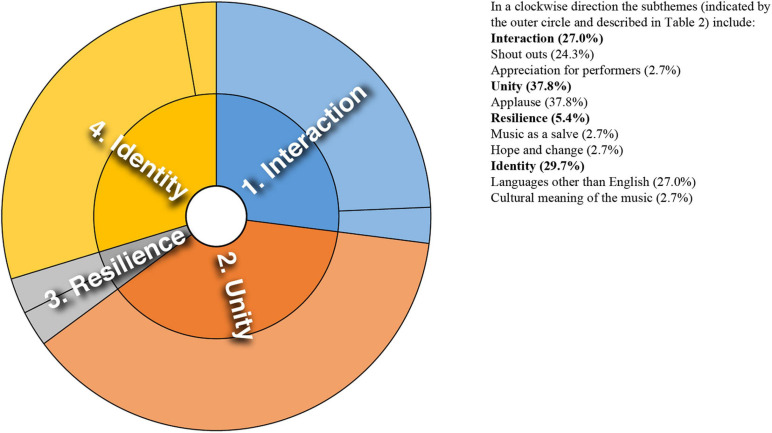
Sunburst diagram of thematic analysis for case study 7, indicating proportion of data for each theme (inner circle) and subtheme (outer circle).

**FIGURE 8:**
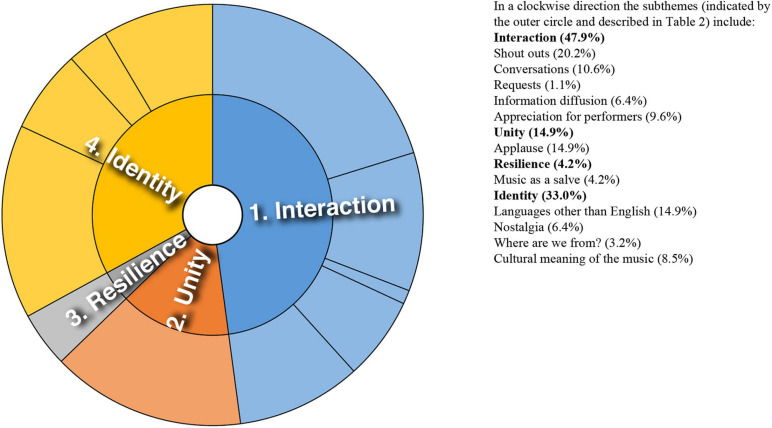
Sunburst diagram of thematic analysis for case study 8, indicating proportion of data for each theme (inner circle) and subtheme (outer circle).

Analysis of converging and diverging themes is included below with a sunburst diagram provided for each case study (see Figures 1–8). The diagrams provide a snapshot of the strength of endorsement of themes for each case. Subthemes that comprise each theme (as described in Table 2) are depicted by the segments in the outer circle of the diagrams and are listed for each case study. As it is not possible to present all variations, the main areas of convergence and divergence are discussed as they apply to the research questions, the literature or emerging themes.

### Case Study 1: Two Performances by Large Scale Community Choir

Case study 1 featured the largest scale performances with community participation. Themes identified for case study 1 are depicted in Figure 1. Emerging strongly was the capacity for the online platform to support bridging opportunities. Weak online ties appeared to facilitate information diffusion and exchanges of advice and emotional support.

Conversations occurred between audience members which appeared to facilitate future participation either with the virtual choir, or as the comment below indicates, the use of the same approach in their own communities. Requests for information about how the choir was directed appeared only for this case study. An example of such an exchange was:

“*Did you use a special app for this or did you record separately and send in? I direct a choir and want to know how to do it*.”

“*They asked us to record ourselves. I used my iPad and sent in the video. The team compiled all the videos*.”

Expressions of support for those voicing loneliness were common and can be summed up by the following exchange:

“*On my own since January but great to see so many people feel the way I do, which is comforting*”

“*Hang in there! I’m on my 16th day, we are all alone, together*”

Coded under the theme *Interaction*, as they represented active audience engagement, these exchanges also relate to the theme of *Resilience* and *sharing lockdown experiences*, a category under *Resilience* that was prominent for this case study. These comments were interpreted as representing community resilience through the exchange of knowledge and emotional support in the context of responding to adversity.

*Information diffusion* was observed via comments indicating the source and subsequent sharing of information. The movement of information through micro, meso and macro levels of the social ecology was revealed by these comments. While sharing the link represents a micro level exchange online, offline this sometimes bridged geographical divides, for example, “This was shared with me in India by friends in Canada.” It was apparent that participants had heard of the choir through traditional media. Traditional media could be considered a macro level, and YouTube could be considered a meso level or bridge, for example, “I saw this on <TV show> and came to YouTube to see it close up.” As the songs were cover versions, they were accompanied by endorsements by the original performers, for example, “The band acknowledged this cover by sharing it.”

Coded under the subtheme *I had a great time* were comments from participants including, “Such a joy and privilege to participate in this choir” and indications of having participated in it with others–“So happy to have been a part of this with my sister.”

*Nostalgia* was a strongly endorsed subtheme under *Identity*. In some cases the music evoked emotional personal memories, for example: “When I was a little girl I had this on a record. When my mom got sick in hospital for a month, I would play this record and cry.” Other comments expressed emotion in the context of the music conjuring the passage of time, for example, “I grew up with this band. Now seeing all the young people in this video fills me with so much emotion.” Such comments also point to the role of music in creating collective identity.

Comments in LOTE including Portuguese, Spanish, German, Japanese, Korean, and Chinese suggested engagement by a culturally diverse audience. The audience commented on the cultural diversity in choir participation, for example “People from so many countries coming together.” One comment expressed dissatisfaction about representation “Not enough African faces.” This contrast, along with a similar pattern in case study 3, suggested an overarching shared identity was important to audiences, but so too was cultural representation.

Unique to case study 1 was the mixture of emotional responses. Most prevalent were comments related to tears, followed by joy. Mixed emotions were specifically expressed. There was an indication of emotional contagion–“Reading “tears of joy” made me burst into tears of joy.” Although isolated, this comment is notable for its explicit demonstration of emotional transfer.

### Case Study 2: Two Remixes of a North African Instrumental Created in Lockdown

Case study 2 was unique in this analysis in that musical collaborations were created organically through shared identification with a cultural form–a vernacular style of music and associated cultural identity, signaled through hashtags and keyword search terms. Emerging themes for case study 2 are depicted in Figure 2. Audience comments were commonly characterized by familiarity with the culture and musical style, and in one case prompted an exchange between audience and producer:

*Audience member: “How did you manage to make that sound like a ribab?”*

*Producer: “Research and observation and also it’s part of my culture.”*

Comments in LOTE were prevalent, similar to case studies 5, 6, 7, and 8. What distinguishes these case studies from case studies 1, 3, and 4 is that they included non-Western musical styles and lyrics sung in LOTE. LOTE were most evident for this case study, with comments predominantly in Arabic, but also French.

Notwithstanding the clustering of Arabic comments in this case study, the presence of comments in other languages highlights the capacity for the online platform to reach a diverse audience, despite the hashtags signaling cultural specificity.

Although the performances were created during lockdown, they did not share the quality of other case studies of being produced as an alternative to live performance. They appeared more representative of new digital forms of cultural participation and practice where audiences become producers through remixing cultural artifacts shared online.

### Case Study 3: South African Song and Dance to Broadcast Health Message

There are many parallels to the themes and subthemes identified for this study and case study 1 (see Figure 3), both cases involving community participation. Expressions of general appreciation for the music were prominent. There were expressions of shared experience–“We are in this together,” and shared identity–“We are a global family.” Although isolated, a counterpoint to this solidarity emerged through a conversation between audience members, likening the performance to propaganda:

*Commenter 1: “Pure propaganda”*

*Commenter 2: “I agree*–*people indoctrinated into complacency”*

*Commenter 3: “What propaganda? Please explain”*

The exchange provided a contrast to the expressions of emotional support and acknowledgment of shared experience evident in case study 1.

Audience members expressed a sense of nostalgia and identification, for example, “A song from my childhood,” and comments referred to the original composer and cultural origins of the song. Similarly to case study 1, there were both positive and negative comments about the representation of cultural diversity in the video. For example, “I like that many different African countries are shown, while there is no clip from countries which would dominate Western news,” and conversely, “I don’t see Côte d’Ivoire there.”

Emotional responses to the video were all characterized by joy or suggesting a humorous engagement, for example “Very funny.” Comparable comments were evident in case study 1 and may have been related to the community participation, with some individual submissions emphasizing humor and play.

### Case Study 4: North American and East Asian Orchestra Collaboration

In this case study, like case studies 1 and 6, music was expressed as a way to “unite the world.” Figure 4 provides an overview of the themes. Appreciation directed toward the musicians was prominent in the comments. Sadness was conveyed at the loss of opportunities to experience live music. Referring to the venue the North American orchestra would usually perform in, one audience member commented:

“*It is beyond sad when I walk by the venue. We are all waiting for it to come alive again with performances and audiences. Until then, thank you for these virtual performances*.”

There were only comments in English and the language of the East Asian orchestra, although comments in English indicated audience participation from English speaking countries other than North America.

### Case Study 5: World Fusion Music Collaboration–Charity Raising Effort

The subthemes emerging from the coding of case study 5 were similar to case studies 1 and 3, despite the lack of community participation in this case (see Figure 5). However, footage of disaster response efforts within communities may account for these converging themes, as well as the participating musicians themselves appearing to represent a community of like-minded artists with a select fan base.

Indeed, this case study featured brief responses by the broadcasting musician to comments by the audience, highlighting the capacity for direct connection between musicians and their fans, which in this case revealed some exclusivity in the engagement. A comment by one of the performers, “So honored to take part in this noble cause” further highlighted the permeable boundary between performer and audience in online platforms.

Emotion was expressed, which together with case studies 1, 3 and 4, suggest a propensity for at least some audience members to experience emotional responses despite the mediated form of engagement with the music.

### Case Study 6: Asia Minor Informal Orchestra

This case study was similar to case study 2, also featuring a culturally specific vernacular style of music. As discussed in case study 2, this might account for the prevalence of comments in LOTE, predominantly Greek and Turkish. However, like case study 5, connections appear to have been facilitated through informal association with an existing community of musicians rather than finding affiliation online via hashtags, which did not feature in the introductory texts for this case study. Themes identified for this case study are depicted in Figure 6.

Cultural diversity in music participation was observable through the video and the nationalities identified in the introductory text, but this was not commented on by audience participants. However one comment, made by a participant of the orchestra, acknowledged the effort of the Southern Hemisphere organization in facilitating a bridge between musicians located in the Northern and Southern Hemispheres.

The notion that *music unites*, overlapping with the theme of *Resilience* was encapsulated by the comment, “Musicians coming together across our troubled world.” Also coded under *Resilience* were comments suggesting participation in the performance brought hope, with some comments by participating musicians praising the conductor, for example, “you have been a catalyst and an inspiration.”

This engagement as both audience and participant has been discussed previously as emerging in case studies 1 and 5. This case study is more similar in nature to case study 5 than case study 1 with regards to the blurred lines between audience and participation. Although the musicians appeared part of a community rather than a formal orchestra like case study 4, they were nonetheless proficient musicians, rather than amateurs sourced from the broader community. As such, comments that personally addressed other participants were characterized by a certain exclusivity observable in groups reinforced by bonding capital. Indeed participation in this performance would have been predicated on knowledge of the particular musical style and audience comments sometimes simply stated the name of the style in the comments, seemingly as a way to signal recognition.

### Case Study 7: West African/Arabic Trio

This case study (see Figure 7) continues a theme emerging from case studies 5 and 6 of comments suggesting engagement by a select fan base, with the broadcasting musician addressed by name on several occasions. Comments directed to the broadcasting musician, such as “Your music is a great healer” were acknowledged by the musician with a “love” reaction.

As has been discussed in case studies 2, 5 and 6 and likewise for the final case study that follows, comments in LOTE were prevalent. The performance was identified as an Islamic invocation in its title and introductory texts and the performance took place in West Africa and Western Europe. This confluence appeared to attract a culturally diverse audience, with LOTE including Arabic, French, Portuguese, Malagasy, Spanish, and Nyanja. The cultural significance of the music was discussed in terms of the use of traditional instruments, and vocalization in an Arabic style, for example, “Such sensitive playing on the kora.” Like case studies 2, 5, and 6, such comments suggest engagement by audiences familiar and interested in the musical genre performed.

### Case Study 8: West African/Western Classical Duo

This case study shares many subthemes under the theme *Interaction* with case studies 1 and 5 (see Figure 8). Case study 1 featured community participation and case study 5 featured audience engagement suggestive of a select fan base. Interestingly case study 8 featured two very well-known artists where there were many comments expressing love for the artists, addressing them by name. However, for this case, unlike case study 5, there were no exchanges between musicians and fans.

Comments in LOTE, included Portuguese, French, Nyanja and Spanish, suggesting diverse cultural engagement. A conversation ensued, exchanging knowledge about the dialect in which the song was sung:

*Commenter 1: “The language is <dialect >, am I right?”*

*Commenter 2: “The song originates from < place >. Here a language close to <dialect 1> is called <dialect 2>”*

*Commenter 3: “So is she singing in <dialect 2>?”*

Similar to the data for case studies 1 and 3, such exchanges suggested representation and acknowledgment of difference was important to cultural identity.

While some comments suggested personal identification with the music, such as, “I learned this song when I was 7 years old in Senegal,” others indicated exposure to new cultural experiences, such as “I don’t understand a word, but it’s so soothing.” The latter example illustrated the way music, as a non-verbal medium, can overcome language barriers, with potential to facilitate intercultural understanding.

## Discussion

The research aimed to investigate whether online music engagement facilitated social cohesion, intercultural understanding and community resilience during COVID-19 lockdown. Specifically, the study sought to address: (a) how audiences engaged with online music broadcasts as ritual; (b) the role played by online music ritual in engendering shared identity and the mechanisms implicated; (c) the interaction between different cultural identities as part of online music engagement; (d) the factors influencing dissemination of the videos; and (e) the way in which community resilience was enacted through online music engagement. Given the idiographic nature of this research, the results above offer an account of the potential social outcomes experienced by particular online communities during their engagement with music during COVID-19 lockdown. Rather than producing broad inferences, this research offers insight into the potential for connection in online spaces at a time when online spaces where all that were available to many around the world.

### Engagement With Online Music Broadcasts as Ritual

Five themes emerged from coding of the data including *Interaction*, *Unity*, *Resilience*, *Identity*, and *Emotion*. These themes are consistent with psychosocial effects observed of ritualized collective gatherings in offline settings where emotional communion has been observed to strengthen collective identity, identity fusion, enhancement of personal and collective efficacy and positive social beliefs (Páez et al., 2015). However, contrary to offline settings, emotional communion did not appear to be prominent in this study. The phenomenon of emotional communion is associated with synchronized behavior and shared experience *in vivo*. For the asynchronous engagement observed in these case studies, Couldry’s (2003) interpretation of Durkheim’s ritual theory of social integration, with its emphasis on collective knowledge over collective feeling is instructive. Couldry defined collective knowledge as “the cognitive processes and categorizations (inevitably more dispersed across space and not requiring us to congregate in one place) on which our knowledge of the social world is based” (p.22).

The ritualized nature of audience engagement with music broadcast via YouTube during the COVID-19 pandemic was characterized by positive group responses directed at the performance or performers and signaling of location around the world. Other ritual actions such as the use of hashtags or specific styles of online discourse (Zappavigna, 2012) were not pronounced in the case studies selected. Case study 2 was a notable exception, featuring the use of hashtags and keyword search terms to signal affiliation with a specific cultural group and musical style. Case study 2 also diverged from the other case studies in the way the performances did not seem to represent an alternative to live performance. Rather the remixing of cultural products shared online represented emerging digital forms of cultural participation which blur the boundaries between cultural consumer and producer. The apparent collective ownership of a cultural product calls to mind Baym’s (2007) idea of the “networked collectivism,” and more democratic and participatory models of cultural production online discussed by commentators including Benkler (2006) and Mishra and Henriksen (2018). As engagement with cultural artifacts online is predicated on knowledge of a cultural form, it is conceivable that cognitive mechanisms would be predominant.

### Engendering Shared Identity and Implicated Mechanisms

Expressions of unity were observed, with references to shared humanity and music’s specific role in uniting people. The shared experience of the pandemic, and appreciation for musical performances also represented collective responses.

As discussed, although these were not prominent, comments by some audience members suggested an emotional response to the broadcasts that may have served to engender a sense of shared identity. However, there is insufficient data in this analysis to draw conclusions about the role of emotion in strengthening collective identity, or to surmise whether empathy played a role, as suggested by previous literature (Vuoskoski and Eerola, 2012; Vuoskoski et al., 2017; Rosenbusch et al., 2019).

Identification with the music through a sense of nostalgia emerged for half of the case studies. Reminiscence and nostalgia through music listening as a form of social surrogacy has been associated with social cognition and connectedness, through bringing back memories of significant people or events (Schäfer and Eerola, 2020). In this analysis, the memories evoked for some people were personal in nature, for others they communicated a sense of being part of a collective. What is interesting in this analysis, particularly in the context of music listening as a social surrogate, is the affordance provided by the YouTube platform to share personal memories with other users, which likewise potentially creates a sense of connectedness.

Representations of cultural diversity through the videos were noted positively by audiences, with the potential to facilitate future intercultural interactions. The affordances created by online platforms for interactions between audience members facilitated exchange of knowledge about specific cultural musical forms, providing opportunity for social learning.

A degree of homophily was apparent in certain case studies supporting more exclusive engagement, particularly those presenting vernacular styles of music associated with a specific cultural group. Despite this relative exclusivity suggestive of bonding capital (Coleman, 1988; Putnam, 2000), the range of languages represented in the comments for these case studies pointed to some cultural diversity in engagement, supporting Benkler’s (2006) assertion of the prevalence of bridging opportunities online.

### Interaction Between Different Cultural Identities

Although language may have been a barrier to bridging between different cultural identities via dialog, music appeared to emerge as a non-verbal medium that facilitated diverse engagement, an observation made in offline intercultural music engagement (Vougioukalou et al., 2019). Exchanges about specific cultural knowledge including the use of traditional instruments, styles of vocalization and use of specific dialects revealed interaction between different cultural identities.

Music, representation and acknowledgment of differences all emerged as being important to cultural identity. It has been observed in the literature that the importance of acknowledging diverse cultural identities has implications for research and policy development in the areas of social cohesion (Abascal and Baldassarri, 2015), community resilience (Grossman, 2014), and for the way music studies are conducted (Jacoby et al., 2020). The representation in the videos of transnational identities and diasporic communities underlines the complexity of culture and intercultural understanding in the context of globalization and widespread use of information and communications technology.

### Factors Influencing Dissemination of the Videos

A number of factors appeared to influence dissemination of videos including individual shares, endorsements by opinion leaders, traditional media and organizations, consistent with previous literature (Sajuira et al., 2015; Hilbert et al., 2017). Micro, meso and macro level processes appeared to intersect as part of a dynamic system, where individual shares could bridge geographic location, and information diffusion through traditional media led to online engagement. The YouTube platform emerged as an important bridge connecting musicians and producers with a wider audience.

The capacity to engage in dialog through the comments function also served to blur the divide between performer and audience, creating affordances for a range of levels of interaction, including in the case of a large-scale community choir, the possibility to participate in future online performances. This has implications for community resilience, discussed further below, but also for further dissemination of the videos through individual shares by participants themselves.

### How Community Resilience Was Enacted Through Online Music Engagement

Well-known and lesser-known musicians, formal and informal choirs, orchestras and music groups broadcast via YouTube, used music for a range of purposes including charity raising efforts, morale boosting, broadcasting health related messages, and engaging global community participation. The use of music for all of these purposes has implications for building community resilience during lockdown. Acknowledgment of the shared experience of adversity, positive collective responses and interaction between audience participants represented displays of community resilience.

### Conclusion

Both Hesmondhalgh (2013) in his analysis of the role of music in fostering collective identity and Couldry (2003) in his analysis of media rituals, referred to John Durham Peters book *Speaking into the air*, which challenged the notion that face-to-face is the only legitimate form of communication. Indeed, in this study, the sharing of music videos and online dialog appeared to create opportunity for meaningful exchange. Weak online ties translated into emotional support and sharing of knowledge. The data pointed to a shared sense of identity through both experiencing the pandemic and feeling buoyed by the music, but also to the importance of specific cultural knowledge and representation.

### Limitations and Future Directions

A limitation of the study was that the data is idiographic in nature due to the ethnographic approach. Having been drawn from videos shared with the first author, from others actively engaged in intercultural music and dance, it is possible that the degree of cultural diversity of engagement is a function of the social network through which the data was sourced, itself representing a form of homophily–a shared interest in music of diverse cultures. While audience comments were suggestive of the emergence of social connections, intercultural dialog and resilient responses, the observations are not broadly generalizable. The study nonetheless points to the possibilities for bridging, intercultural understanding and cohesion afforded by the online environment through music engagement.

As an exploratory study, although the unobtrusive mode of data collection facilitated observation of social processes in a naturalistic setting, it precluded deeper understanding of the experience and motivations of participants. Future research that integrates alternate forms of data collection is necessary to better support extrapolation of findings.

## Data Availability Statement

The datasets presented in this article are not readily available because the data are not publicly available due to them containing information that could compromise research participant privacy. Requests to access the datasets should be directed to TF, trisnasari.fraser@unimelb.edu.au.

## Ethics Statement

The studies involving human participants were reviewed and approved by University of Melbourne Human Research Ethics Committee. Written informed consent for participation was not required for this study in accordance with the national legislation and the institutional requirements.

## Author Contributions

TF conceived and designed the study, carried out the literature review, collected and analyzed the data, and wrote the manuscript. AC and JWD provided critical feedback on the data analysis. All authors contributed to manuscript revisions, approved the final version of the manuscript and agreed to be accountable for the content herein.

## Conflict of Interest

The authors declare that the research was conducted in the absence of any commercial or financial relationships that could be construed as a potential conflict of interest.
